# Supplementary material for: A Longitudinal Study on Cognitive Training for Cognitively Preserved Adults in Liguria, Italy
Source: Healthcare (Basel). 2024 Feb 2;12(3):393. doi: 10.3390/healthcare12030393 (PMC10855271; doi:10.3390/healthcare12030393)
Supplement: Supplementary file 1 [file healthcare-12-00393-s001.zip › healthcare-2787183-supplementary.pdf]

Table S1. Assumption test Results of Regression Model on time T0

	**Linearity test (F test)**	**Normality (Shapiro Wilk test)**	**Heteroscedasticity test (scatter plot test)**	**Autocorrelation test (Durbin-Watson test)**	**Multicollinearity test (Variance inflation factors test)**	**Description**
**MACQ**	F test values in the active/passive group, gender, age, and education were <0.0001, <0.0001, 0.7415, and 0.7567 respectively	<0.0001	The points did not form a particular pattern and spread	0.8953	VIF values of diversification in the active/passive group, gender, age, and education were 1.0004, 1.0012, 1.3819, and 1.3831 respectively <10	Passed linearity test for group, and gender variables; data were not normally distributed; heteroscedasticity did not occur; No autocorrelation; multicollinearity did not occur
**GDS-4**	F test values in the active/passive group, gender, age, and education were <0.0001, 0.2483, 0.0152, and <0.0001 respectively	<0.0001	The points did not form a particular pattern and spread	1.9384	VIF values of diversification in the active/passive group, gender, age, and education were 1.0004, 1.0012, 1.3819, 1.3831 respectively <10	Passed linearity test for group, age, and education variables; data were not normally distributed; heteroscedasticity did not occur; No autocorrelation; multicollinearity did not occur
**A3LP**	F test values in the active/passive group, gender, age, and education were <0.0001, 0.9349, <0.0001, and <0.0001 respectively	0.0003	The points did not form a particular pattern and spread	1.490	VIF values of diversification in the active/passive group, gender, age, and education were 1.0004, 1.0012, 1.3819, 1.3831 respectively <10	Passed linearity test for group, age, and education variables; data were not normally distributed; heteroscedasticity did not occur; No autocorrelation; multicollinearity did not occur
**VF**	F test values in the active/passive group, gender, age, and education were 0.0477, 0.3647, <0.0001, and <0.0001 respectively	0.0006	The points did not form a particular pattern and spread	1.8452	VIF values of diversification in the active/passive group, gender, age, and education were 1.0004, 1.0012, 1.3819, 1.3831 respectively <10	Passed linearity test for group, age, and education variables; data were not normally distributed; heteroscedasticity did not occur; No autocorrelation; multicollinearity did not occur

Table S2. Assumption test Results of Regression Model on time T1

	**Linearity test (F test)**	**Normality (Shapiro Wilk test)**	**Heteroscedasticity test (scatter plot test)**	**Autocorrelation test (Durbin-Watson test)**	**Multicollinearity test (Variance inflation factors test)**	**Description**
**MACQ**	F test values in the active/passive group, gender, age, and education were <0.0001, 0.1719, 0.2184, and 0.3185 respectively	0.0221	The points did not form a particular pattern and spread	1.4564	VIF values of diversification in the active/passive group, gender, age, and education were 1.0004, 1.0012, 1.3819, and 1.3831 respectively <10	Passed linearity test for group variable; data were not normally distributed; heteroscedasticity did not occur; No autocorrelation; multicollinearity did not occur
**GDS-4**	F test values in the active/passive group, gender, age, and education were <0.0001, 0.1520, 0.0220, and 0.0037 respectively	<0.0001	The points did not form a particular pattern and spread	2.0216	VIF values of diversification in the active/passive group, gender, age, and education were 1.0004, 1.0012, 1.3819, 1.3831 respectively <10	Passed linearity test for group, age, and education variables; data were not normally distributed; heteroscedasticity did not occur; No autocorrelation; multicollinearity did not occur
**A3LP**	F test values in the active/passive group, gender, age, and education were <0.0001, 0.3353, <0.0001, and <0.0001 respectively	<0.0001	The points did not form a particular pattern and spread	1.2945	VIF values of diversification in the active/passive group, gender, age, and education were 1.0004, 1.0012, 1.3819, 1.3831 respectively <10	Passed linearity test for group, age, and education variables; data were not normally distributed; heteroscedasticity did not occur; No autocorrelation; multicollinearity did not occur
**VF**	F test values in the active/passive group, gender, age, and education were <0.0001, 0.3904, <0.0001, and <0.0001 respectively	<0.0001	The points did not form a particular pattern and spread	1.8515	VIF values of diversification in the active/passive group, gender, age, and education were 1.0004, 1.0012, 1.3819, 1.3831 respectively <10	Passed linearity test for group, age, and education variables; data were not normally distributed; heteroscedasticity did not occur; No autocorrelation; multicollinearity did not occur

Table S3. Assumption test Results of Regression Model on time T2

	**Linearity test (F test)**	**Normality (Shapiro Wilk test)**	**Heteroscedasticity test (scatter plot test)**	**Autocorrelation test (Durbin-Watson test)**	**Multicollinearity test (Variance inflation factors test)**	**Description**
**MACQ**	F test values in the active/passive group, gender, age, and education were <0.0001, <0.0511, 0.5030, and 0.3753 respectively	<0.0001	The points did not form a particular pattern and spread	1.4496	VIF values of diversification in the active/passive group, gender, age, and education were 1.0004, 1.0012, 1.3819, and 1.3831 respectively <10	Passed linearity test for group and gender variables; data were not normally distributed; heteroscedasticity did not occur; No autocorrelation; multicollinearity did not occur
**GDS-4**	F test values in the active/passive group, gender, age, and education were <0.0001, 0.4298, 0.0793, and 0.0117 respectively	<0.0001	The points did not form a particular pattern and spread	1.1958	VIF values of diversification in the active/passive group, gender, age, and education were 1.0004, 1.0012, 1.3819, 1.3831 respectively <10	Passed linearity test for group and education variables; data were not normally distributed; heteroscedasticity did not occur; No autocorrelation; multicollinearity did not occur
**A3LP**	F test values in the active/passive group, gender, age, and education were <0.0001, 0.5759, 0.0002, and <0.0001 respectively	<0.0001	The points did not form a particular pattern and spread	1.2261	VIF values of diversification in the active/passive group, gender, age, and education were 1.0004, 1.0012, 1.3819, 1.3831 respectively <10	Passed linearity test for group, and education variables; data were not normally distributed; heteroscedasticity did not occur; No autocorrelation; multicollinearity did not occur
**VF**	F test values in the active/passive group, gender, age, and education were <0.0001, 0.6830, <0.0001, and <0.0001 respectively	<0.0001	The points did not form a particular pattern and spread	1.8437	VIF values of diversification in the active/passive group, gender, age, and education were 1.0004, 1.0012, 1.3819, 1.3831 respectively <10	Passed linearity test for group, age, and education variables; data were not normally distributed; heteroscedasticity did not occur; No autocorrelation; multicollinearity did not occur

Table S4. Assumption test Results of Regression Model on time T3

	Linearity test (F test)	Normality (Shapiro Wilk test)	Heteroscedasticity test (scatter plot test)	Autocorrelation test (Durbin-Watson test)	Multicollinearity test (Variance inflation factors test)	Description
**MACQ**	F test values in the active/passive group, gender, age, and education were <0.0001, 0.0058, 0.6089, and 0.6741 respectively	<0.0001	The points did not form a particular pattern and spread	1.5167	VIF values of diversification in the active/passive group, gender, age, and education were 1.0005, 1.0013, 1.3825, and 1.3838 respectively <10	Passed linearity test for group and gender variables; data were not normally distributed; heteroscedasticity did not occur; No autocorrelation; multicollinearity did not occur
**GDS-4**	F test values in the active/passive group, gender, age, and education were 0.0175, 0.9276, 0.0324, and 0.0007 respectively	<0.0001	The points did not form a particular pattern and spread	1.3086	VIF values of diversification in the active/passive group, gender, age, and education were 1.0005, 1.0013, 1.3825, and 1.3838 respectively <10	Passed linearity test for group and education variables; data were not normally distributed; heteroscedasticity did not occur; No autocorrelation; multicollinearity did not occur
**A3LP**	F test values in the active/passive group, gender, age, and education were <0.0001, 0.6560, 0.0040, and <0.0001 respectively	<0.0001	The points did not form a particular pattern and spread	1.2327	VIF values of diversification in the active/passive group, gender, age, and education were 1.0005, 1.0013, 1.3825, and 1.3838 respectively <10	Passed linearity test for group, age, and education variables; data were not normally distributed; heteroscedasticity did not occur; No autocorrelation; multicollinearity did not occur
**VF**	F test values in the active/passive group, gender, age, and education were 0.1785, 0.9706, <0.0001, and <0.0001 respectively	<0.0001	The points did not form a particular pattern and spread	1.7884	VIF values of diversification in the active/passive group, gender, age, and education were 1.0005, 1.0013, 1.3825, and 1.3838 respectively <10	Passed linearity test for age, and education variables; data were not normally distributed; heteroscedasticity did not occur; No autocorrelation; multicollinearity did not occur
